# Is the Temporomandibular Joints’ Reciprocal Clicking Related to the Morphology and Position of the Mandible, as Well as to the Sagittal Position of Lower Incisors?—A Case-Control Study

**DOI:** 10.3390/ijerph18094994

**Published:** 2021-05-08

**Authors:** Marcin Derwich, Maria Mitus-Kenig, Elzbieta Pawlowska

**Affiliations:** 1 ORTODENT, Specialist Orthodontic Private Practice in Grudziadz, 86-300 Grudziadz, Poland; 2 Department of Experimental Dentistry and Prophylaxis, Medical College, Jagiellonian University in Krakow, 31-008 Krakow, Poland; maria.mitus@interia.pl; 3 Department of Orthodontics, Medical University of Lodz, 90-419 Lodz, Poland; elzbieta.pawlowska@umed.lodz.pl

**Keywords:** temporomandibular joint, reciprocal clicking, anterior disc displacement with reduction, temporomandibular joint disorders, internal derangement, cephalometry

## Abstract

The number of patients diagnosed with temporomandibular joint (TMJ) internal derangements, who are seeking orthodontic treatment, is increasing. The aim of the study was to assess the relationship between the presence of TMJ reciprocal clicking and the morphology and position of the mandible, and position of lower incisors, examined on the lateral cephalograms. Fifty patients diagnosed with reciprocal clicking in at least one of the TMJs and 55 patients with no symptoms of TMJ dysfunction were included in the study. Cephalometric analysis was used for the assessment of: skeletal class, sagittal and vertical position of the mandible, angle of the mandible, inclination of the mandibular ramus and the mandibular corpus, as well as for the assessment of the position of the mandibular incisors. The statistical significance level was set at *p* = 0.05. There were no statistically significant differences between the examined groups regarding the sagittal and vertical position of the mandible, as well as regarding the sagittal position of the mandibular incisors. Presence of TMJ reciprocal clicking is not associated with the position and the morphology of the mandible, as well as with the sagittal position of the mandibular incisors. Patients with early stages of TMJ internal derangements do not present any significant changes in Cephalometrics. Patients diagnosed with TMJ internal derangements before orthodontic treatment require an interdisciplinary approach to the treatment, including physiotherapy.

## 1. Introduction

Temporomandibular joint (TMJ) reciprocal clicking is a pathological double-clicking sound localized in the area of the temporomandibular joints (TMJs), which occurs during both mouth opening and closing and is related to the changes in the position of the articular disc. Reciprocal clicking is one of the clinical symptoms typical for the disc displacement with reduction (DDwR) [1,2,3,4,5,6]. DDwR is characterized by the malposition of the articular disc in relation to the mandibular condyle in closed mouth position and by its repositioning during mouth opening [1].

DDwR is one of the most common types of TMJ internal derangements [7,8]. The term “internal derangements” encompasses all of the clinical states in which the position of the articular disc has become disturbed, including, in addition to the previously mentioned DDwR, disc displacement with intermittent locking, disc displacement without reduction (DDwoR) with limited opening, DDwoR without limited opening and posterior disc displacement [7]. It has been estimated that the frequency of disc displacement in the general adult population ranges from 18% to 35% [8], with a higher prevalence in women [8,9]. Anterior disc displacement with reduction (ADDR) also occurs among children (7.0% of children aged 7–12 years), adolescents (14.4% of adolescents aged 7–12 years) and young adults (12.3% of young adults aged 19–21 years) [10]. Moreover, disc displacements have been found to be associated with the degenerative joint disease (DJD). Half of the patients with disc displacement developed DJD. There was higher prevalence of DJD in patients with DDwoR (66%) than in patients with DDwR (35%) [11].

The etiology of internal derangements is contentious [12]. There have been several different factors described which may lead to the displacement of the articular disk, including: anatomical and mechanical factors, trauma, TMJ hypermobility and disturbances in TMJ lubrication [11,12,13,14,15,16]. Nonetheless, none of them appeared to be superior to others [11]. It has also been proven that reducible disc displacements develop most often during earlier life stages (childhood and adolescence) [13].

TMJ internal derangements have been found to be associated with some changes in the morphology of the mandible, including retruded position of the mandible, decreased ramus height and clockwise rotation of the mandible (on the sagittal plane) [17].

Due to the fact that many patients who are seeking orthodontic treatment present symptoms of temporomandibular joint disorders (TMD), including reciprocal clicking [18], it seems necessary to check if the reciprocal clicking is related to the mandibular morphology and its position, and if yes, if can we predict development of TMD in the near future on the basis of cephalometric analysis.

Therefore, the aim of the study was to assess the relationship between the presence of TMJ reciprocal clicking diagnosed during pre-orthodontic extraoral examination and the morphology and position of the mandible, as well as the position of lower incisors, examined on the lateral cephalograms. The null hypotheses are that the TMJ reciprocal clicking is not related to the morphology and position of the mandible nor to the sagittal position of the lower incisors.

## 2. Materials and Methods

This study was approved by The Medical Board Ethical Committee (RNN/74/20/KE) and was conducted with the ethical principles of the World Medical Association Declaration of Helsinki. All of the adult patients and, in case of adolescents, all of their parents received and signed informed consent.

### 2.1. Participants

The study analyzes the same group of patients we have already presented in two of our previous studies [18,19]. There were 50 generally healthy patients included in the study who had been diagnosed with reciprocal clicking with at least one of the temporomandibular joints and 55 generally healthy patients who had never presented any symptoms related to TMD, including reciprocal clicking. All of the participants visited the specialist orthodontic private practice in Grudziadz (Poland) looking for an orthodontic consultation. Patients were allocated into one of the previously mentioned groups on the basis of the anamnesis and the clinical examination of TMJs. Patients did not present any parafunctions. 

Table 1 presents inclusion and exclusion criteria for both groups.

### 2.2. Protocol 

Standard orthodontic examination was performed in all patients who had come to the specialist orthodontic private practice in Grudziadz (Poland) for consultation. The orthodontic diagnosis was formed on the basis of the results of the extraoral and intraoral examination and plaster casts’ analysis, as well as the analysis of radiographs, including lateral cephalograms and dental panoramic tomograms. The lateral cephalograms were obtained in natural head position. Patients were standing and looking straight ahead into the mirror which was placed in front of the cephalostat. Radiographs were taken on MyRay Hyperion X9 3D (Cefla, Imola, Italy).

#### 2.2.1. TMJ Extraoral Examination 

Extraoral examination of TMJs was performed according to the protocol presented by Marpaung et al. [20]. The examination consisted of two parts: palpable examination and auscultation of the TMJs. TMJs examination was performed during both static and dynamic (mouth opening, closing and lateral movements) positions of the mandible. The stethoscope was used for auscultation of the TMJs. When reciprocal clicking had been diagnosed in at least one of the TMJs, the patient was allocated to the study group.

#### 2.2.2. Lateral Cephalograms’ Analysis 

Analysis of lateral cephalograms included different measurements originating from various cephalometric analyses, including Steiner, Jacobson, Tweed, Jarabak and Arnett [21,22,23,24,25,26]. Cephalometric analyses were performed with the software Ortodoncja 9.0 (Ortobajt, Wroclaw, Poland). 

Table 2 presents the list of points, lines and angles used to analyze the mandibular position and morphology, as well as the position of lower incisors on the basis of the literature [21,22,23,24,25,26].

**Figure 1:**
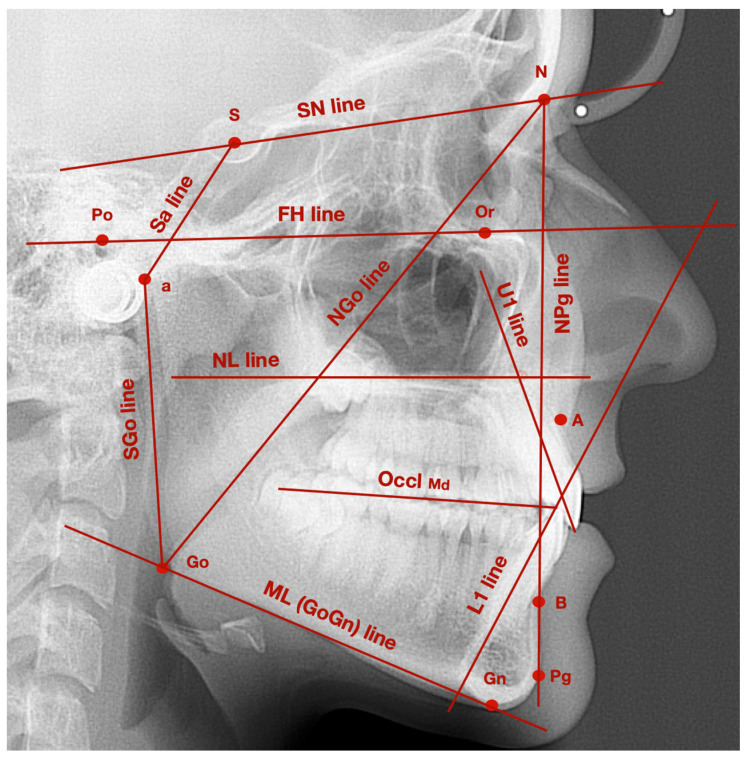
Exemplary lateral cephalogram with marked points and lines described in Table 2.

Figure 1 presents exemplary lateral cephalogram with marked points and lines described in Table 2.

**Figure 2:**
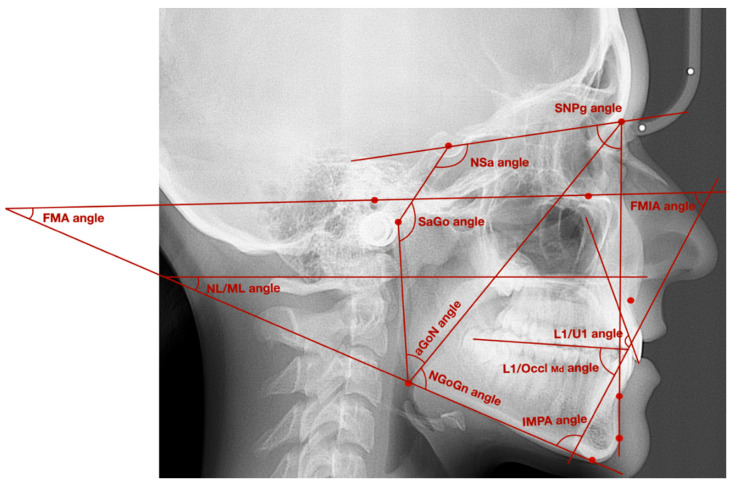
Exemplary lateral cephalogram with marked angles described in Table 2.

Figure 2 presents exemplary lateral cephalogram with marked angles described in Table 2. 

Table 3 presents the list of different cephalometric measurements used for the analysis of: skeletal class, sagittal and vertical position of the mandible, angle of the mandible, inclination of the mandibular ramus and the mandibular corpus, as well as for the analysis of the mandibular incisors position.

### 2.3. Statistical Analysis 

We used Statistica 13.0 software (Dell Inc., Aliso Viejo, CA, USA) to perform statistical analysis. Quantitative variables were characterized with the usage of mean values, standard deviation, range, median and 95% confidence interval. To check if there were statistically significant differences between the examined groups, the following tests were used: T-Student test, U Mann‒Whitney, Chi-square test. The statistical significance level was set at *p* = 0.05.

## 3. Results

The sample size was estimated based on an initial pilot study that analyzed the morphologic difference between the groups. A total of a maximum 50 patients were necessary in order to detect a 10% difference between the parameters with a power of 80% and probability of the type I error 0.05.

### 3.1. Demographic Characteristics of the Examined Patients 

A total of 105 patients took part into the study. These patients were allocated into one of the two groups on the basis of the presence of reciprocal clicking in at least one of the TMJs. The study group consisted of 50 patients (12 males, 38 females; median age: 21.75; range: 16–47), who had been diagnosed with reciprocal clicking. The control group consisted of 55 generally healthy patients (14 males and 41 females; median age: 22.70; range 16–44), who had never presented any symptoms related to TMD, including reciprocal clicking. There were no statistically significant differences between the groups regarding both age and sex. 

Table 4 presents a comparison of age and sex between the examined groups.

### 3.2. Skeletal Class Assessment 

The majority of patients in both groups were diagnosed with skeletal class II (46% of patients in the study group vs. 43.64% in the control group). Skeletal class I was diagnosed in 32% of patients in the study group and 41.82% of patients in the control group). Skeletal class III was diagnosed least frequently, namely 22% of patients with TMJ reciprocal clicking and 14.54% of patients with no symptoms of TMD. There were no statistically significant differences in the distribution of skeletal classes between the examined groups.

Table 5 presents the distribution of skeletal classes between the examined groups.

### 3.3. Assessment of the Position and Morphology of the Mandible

There were no statistically significant differences between the examined groups regarding the sagittal position of the mandible on the basis of the following angles: SNPg, NSa and SaGo. 

The average FMA value in patients diagnosed with reciprocal clicking was 25.3° (4.9°), whereas in the control group the average FMA value was smaller: 24.9° (5.7°). The average NL/ML value was also smaller in the control group compared to the study group. Although patients diagnosed with reciprocal clicking presented increased an vertical dimension compared to the control group, the differences between the groups were not statistically significant.

There were no statistically significant differences between the examined groups regarding the average value of the angle aGoGn (gonial angle), the angle aGoN (upper gonial angle) and the angle NGoGn (lower gonial angle). The average value of the angle aGoN in the study group was 49.5° (3.9°), whereas in the control group it was 51.0° (4.4°). The average value of the angle NGoGn (lower gonial angle) in the group of patients with TMJ reciprocal clicking was 72.5° (5.3°), whereas in the control group it was 71.6° (5.1°). 

Table 6 presents the assessment of the position and morphology of the mandible in both groups.

### 3.4. Position of the Mandibular Incisors 

No statistically significant differences regarding the angles FMIA, IMPA, L1/Occl_Md_ and L1/U1 were found between the examined groups.

Table 7 presents the assessment of the position of the mandibular incisors in both groups. 

## 4. Discussion

It has been proven that temporomandibular disorders, including anterior disc displacement, have a negative effect on oral health-related quality of life, mostly because of physical pain, physical disability and psychological discomfort [27,28,29].

According to our knowledge, this is the first study that analyzes the morphology and the position of the mandible, as well as the sagittal position of lower incisors with reference to the presence of the reciprocal clicking in the temporomandibular joints.

We have not found any significant differences regarding both the sagittal and vertical position of the mandible between the examined groups. The average values of different angles describing the sagittal (SNPg, NSa, SaGo) and vertical (FMA, NL/ML) position of the mandible were within the reference values [21,22,23,24,25,26]. However, the mean value of the SNPg angle was slightly decreased and the mean value of the SaGo angle was slightly increased in patients diagnosed with TMJ reciprocal clicking compared to the asymptomatic patients. These changes indicate a slightly retruded position of the mandible in the “reciprocal clicking” group. Moreover, the mean values of FMA and NL/ML angles were also slightly increased in patients with TMJ reciprocal clicking, probably indicating a tendency towards a vertical growth pattern in patients with more advanced internal derangements. The average value of the upper gonial angle was decreased and the average value of the lower gonial angle was increased in patients with reciprocal clicking. Although the differences between the groups were statistically insignificant, reduced values of the upper gonial angle are typical for clockwise rotation of the mandible and a vertical growth pattern. This remains in accordance with slightly increased values of FMA and NL/ML angles in “reciprocal clicking” patients. We also did not find any significant differences regarding the sagittal position of mandibular incisors between the examined groups. Nonetheless, patients with clinical symptoms of internal derangements presented an increased average value of the interincisal angle, L1/Occl_Md_ angle and FMIA angle, as well as a decreased average value of the IMPA angle. These results indicate a tendency toward a more retroclined position of the lower incisors in patients with “reciprocal clicking”. It must be emphasized once again that all of our patients diagnosed with TMJ reciprocal clicking did not present any symptoms of irreducible disc displacement nor had they ever had any history of mandibular intermittent locking or limited mouth opening. Thus, all of the symptomatic patients included into the study presented initial symptoms of internal derangements. This could explain why the differences between the study and the control groups were not statistically significant.

Gidarakou et al. [30,31,32,33] published a series of four manuscripts, where they analyzed skeletal and dental changes in female patients with different types of internal derangements: unilateral disc displacement with reduction (18 women) [30], bilateral disc displacement with reduction (42 women) [31], unilateral disc displacement without reduction (12 women) [32] and bilateral disc displacement without reduction (59 patients) [33]. The authors compared each of the previously listed groups with the asymptomatic group of female volunteers (46 women). Patients diagnosed with unilateral disc displacement with reduction presented significantly reduced length of the anterior and posterior cranial base, increased cranial base angle, retroposition of both jaws (reduced SNA and SNB angles) and significant reduction of the posterior ramal height [30]. Females with confirmed bilateral disc displacement with reduction presented, similarly to the previous group, significantly smaller length of both anterior and posterior cranial base and decreased SNA and SNB angles. In addition to this, they were also diagnosed with a significantly increased interincisal angle and more retroclined upper incisors [31]. Interestingly, although bilateral disc displacement with reduction is a more severe type of internal derangement compared to the unilateral one, this group did not present any significant differences related to the length of the posterior ramal height. Even though the mean values of the measurements related to the vertical dimension were slightly increased in both groups compared to the healthy volunteers, the differences were not statistically significant [30,31]. These two groups may be compared to our study group, because reciprocal clicking is a typical, clinical symptom of reducible disc displacement, also known as disc displacement with reduction. Gidarakou et al. found that the average value of the SNB angle was significantly lower in both of the examined groups. Our results are not in accordance with that observation. We only noticed a slight tendency, but statistically insignificant, toward a decreased value of the SNB angle. This inconsistency may be caused by a different distribution of skeletal malocclusions in patients included in the asymptomatic control group. In our study, 43.64% of patients with no symptoms of internal derangement were diagnosed with skeletal class II. In the studies by Gidarakou et al. [31,32], the average ANB angle was 2.6° (SD = 2.5°). Although Gidarakou et al. did not show the exact distribution of skeletal classes within the examined groups, the presented mean value of the ANB angle (±SD) indicates that the majority of patients in the control group were diagnosed with skeletal class I. On the other hand, both of our studies present the same observations regarding the vertical dimension of patients with early stages of internal derangement.

According to the further studies by Gidarakou et al. [32,33], patients with irreducible disc displacement presented more advanced dentoskeletal changes. Unilateral disc displacement without reduction was associated with a significantly reduced length of the anterior and posterior cranial bases, as well as with shortening of the posterior ramal height, increased facial vertical dimension manifested by increased steepness of the mandibular plane angle and finally with infraeruption of the lower first molars [32]. Patients with bilateral disc displacement without reduction showed a significantly smaller cranial base (but contrary to other groups, there were no differences related to the lengths S-Na and Ba-S), reduced facial plane angle and increased angle of convexity, mandibular retrusion (decreased SNB angle, no significant changes related to the anteroposterior position of maxilla), increased overjet, shortening of the posterior ramal height and an increased vertical dimension (steeper mandibular plane angle and increased angle between mandibular and palatal planes) [33]. Although these studies clearly present cephalometric changes which occurred in patients diagnosed with different stages of internal derangements, it must be emphasized that Gidarakou et al. included only women in the control and study groups. In the literature, estrogen receptor polymorphism has been found to be associated with TMJ osteoarthritis [34,35] and TMJ internal derangements have been linked TMJ osteoarthritis [36,37]. Nearly 25% of patients included in our study (both groups) were men, which could also have had an impact on the final results.

A similar study was conducted by Sakar et al. [38]. The authors also noticed an association between the progression of the articular disc displacement and clockwise rotation of the mandible, decrease in ramus height and decrease in the ratio of posterior to anterior face height. Ahn et al. [39] found that the majority of skeletal changes, including decrease in posterior facial height, decrease in ramus height, clockwise rotation of the mandible, and retruded position of the mandible, were related to the more advanced stages of internal derangement, namely disc displacement without reduction. Although there existed some differences between the control group and patients with disc displacement with reduction, most of them were statistically insignificant. It should be noted that the study by Ahn et al. was based on patients with class II malocclusion. The mean ANB angle in the control group was 5.8° (SD = 1.0°). The authors did not find any statistically significant differences regarding the sagittal position of the mandible between patients with disc displacement with reduction and the control group. The presented results are in accordance with the results obtained in our study.

Bósio et al. [40] found that symptomatic patients diagnosed with TMJ disc displacement occurring bilaterally were characterized by more retruded mandibles. The sagittal position of the mandibles was assessed on the basis of the SNB angle. Unfortunately, because of the fact that the authors did not distinguish between different types of disc displacement, we cannot relate our results to this study. Contrary to the results presented by Gidarakou et al. [30,31,32,33], the authors did not notice any significant differences regarding the cranial base.

Shu et al. [17] conducted a systematic review, in which they concluded that especially the size (smaller ramus height, short mandible) and position (clockwise rotation, retruded position) of the mandible were strongly associated with the presence of TMJ internal derangements.

Differing from the previously presented studies, Brand et al. [41] found that TMJ internal derangements were only related to the reduced lengths of both the maxilla and mandible. The authors did not notice any other significant differences between the examined groups. However, it should be noted that Brand et al. [41] allocated patients with different types of internal derangements to the study group, including: unilateral disc displacement with reduction, unilateral disc displacement without reduction, bilateral disc displacement with reduction and bilateral disc displacement without reduction. As we know from the previously presented studies by Gidarakou et al. [30,31,32,33] and Sakar et al. [38], these four types of internal derangements have been found to be associated with different changes in mandibular position and morphology. Probably if Brand et al. [41] had differentiated those types of internal derangements, they could have reached different results.

Patients diagnosed with TMJ reciprocal clicking require an interdisciplinary approach to the treatment, including physiotherapy and occlusal splint therapy, and could be offered innovative strategies in the field of conservative dentistry [42,43,44].

There are several limitations to our study. Firstly, the study was based mostly on people in their twenties. The average age in both groups was nearly 25 years old. Therefore, to draw general conclusions, similar research should be performed, including with elderly people. Secondly, the number of patients diagnosed with reciprocal clicking was limited to 50 people. This number is comparable to the number of patients included in other studies. However, a larger number of participants would have made the final results more representative. Thirdly, we did not evaluate the diagnosis of reciprocal clicking with magnetic resonance imaging. There are several different positions, where the articular disc can be displaced, not only in the sagittal, but also in the horizontal dimension. Many types of articular disc malposition may lead to the presence of reciprocal clicking. It is worth checking in further studies, if particular types of disc displacement are related to the morphology and position of the mandible. Finally, sometimes internal derangements may be caused by increased masticatory muscle tension or long-term osteoarthritic changes, which have not been discussed within this study. 

## 5. Conclusions

TMJ reciprocal clicking is a typical clinical symptom of disc displacement with reduction, one of the most common type of TMJ internal derangements. Presence of TMJ reciprocal clicking is not associated with the position and the morphology of the mandible or with the sagittal position of the mandibular incisors. Patients with early stages of TMJ internal derangements do not present any significant changes in Cephalometrics. Patients diagnosed with TMJ internal derangements before orthodontic treatment require an interdisciplinary approach to the treatment and should be referred to physiotherapists, and sometimes, especially in case of more severe types of TMJ internal derangements, also to maxillofacial surgeons and even rheumatologists. 

## Figures and Tables

**Table 1:** Inclusion and exclusion criteria for both groups (study group and control group).

Group	Inclusion and Exclusion Criteria for Group Selection
General	*Inclusion criteria:* - generally healthy patients (no systemic diseases) - age between 16 and 60 years old - willingness to participate in the study - no previous orthodontic treatment *Exclusion criteria:* - age below 16 and above 60 years old - TMJ ankylosis - pregnancy - rheumatological diseases, oncological diseases - people who had undergone radiotherapy (especially in the area of head and neck) - patients who had ever had any traumas in the area of head and neck - people treated orthodontically at least once in the past - patients who did not agree to take part in the study
Study group (n = 50 patients)	*Inclusion criteria:* - presence of TMJ reciprocal clicking in at least one of the TMJs *Exclusion criteria:* - other types of internal derangements apart from DDwR
Control group (n = 55 patients)	*Inclusion criteria:* - no symptoms of TMD, no presence of reciprocal clicking *Exclusion criteria:* - any symptoms of TMD

**Table 2:** List of points, lines and angles used to analyze the mandibular position and morphology, as well as the position of lower incisors on the basis of the literature [21,22,23,24,25,26].

Points/Lines/Angles	Description of Points/Lines/Angles
Point S	*Sella*—geometrical center of Sella turcica
Point N	*Nasion*—the most anterior point localized in the frontonasal suture
Point Go	*Gonion*—point localized at the intersection of mandibular line and line tangent to the posterior border of mandibular ramus
Point a	*Articulare*—point localized at the intersection of sphenoid bone base and the posterior part of condylar outline
Point Po	*Porion*—the most superior part of external acoustic opening
Point Or	*Orbitale*—the most inferior point localized in the lower margin of the orbit
Point A	*Subspinale*—the deepest point localized in the anterior outline of the maxilla, below the anterior nasal spine
Point B	*Supramentale*—the deepest point localized in the anterior outline of the mandible, above the pogonion
Wits	*AO-BO*—the distance between the perpendicular projection of points A and B onto the functional occlusal plane
Point Pg	*Pogonion*—the most prominent point localized in the osseous outline of mental tuberosity
NL line	*Nasal line*—line between anterior and posterior nasal spines
ML line	*Mandibular line*—line between gnathion and the lowest point localized in the masseteric tuberosity (also known as GoGn line)
FH line	*Frankfort horizontal line*—line between points: porion and orbitale
L1 line	*Long axis of lower incisor*—line which connects the incisal edge with the radiological apex of lower incisor
U1 line	*Long axis of upper incisor*—line which connects the incisal edge with the radiological apex of upper incisor
Occl_Md_	*Mandibular occlusal plane*—line which connects the anterior cusp tips of lower first molars with the incisal edges of the lower central incisors
SN line	*Sella-nasion line*—line of anterior cranial base, which connects points: nasion and sella
Sa line	Line which connects points: sella and articulare
aGo line	Line which connects points: articulare and gonion
NGo line	Line which connects points: nasion and gonion
NPg line	Line which connects points: nasion and pogonion
SNPg angle	Angle between SN line and NPg line
NL/ML angle	Angle between NL line and ML line
FMA angle	Angle between FH line and ML line
FMIA angle	Angle between FH line and long axis of lower incisor
IMPA angle	Angle between long axis of lower incisor and ML line
L1/U1 angle	*Interincisal angle*—angle between long axes of lower and upper incisors
L1/Occl_Md_ angle	Angle between long axis of lower incisor and mandibular occlusal plane
NSa angle	*Saddle angle*—angle between SN line and Sa line
SaGo angle	*Articular angle*—angle between Sa line and aGo line
aGoGn angle	*Gonial* angle—angle between aGo line and GoGn line
aGoN angle	*Upper gonial angle*—angle between aGo line and NGo line
NGoGn angle	*Lower gonial angle*—angle between NGo line and GoGn line

**Table 3:** List of different cephalometric measurements used for the analysis of: skeletal class, sagittal and vertical position of the mandible, angle of the mandible, inclination of the mandibular ramus and the mandibular corpus, as well as for the analysis of the mandibular incisors position.

**Analysis of Morphology and Position of the Mandible ** **and Position of the Mandibular Incisors**	**Measurement**
Skeletal class	Wits analysis
Sagittal position of the mandible	SNPg angle NSa angle SaGo angle
Vertical position of the mandible	FMA angle NL/ML angle
Angle of the mandible	aGoGn angle
Inclination of mandibular ramus	aGoN angle
Inclination of mandibular corpus	NGoGn angle
Position of the mandibular incisors	FMIA angle IMPA angle L1/Occl_Md_ angle L1/U1 angle

**Table 4:** Comparison of age and sex between the examined groups.

Comparable Characteristic	Study Group (n = 50) “Reciprocal Clicking”	Control Group (n = 55) “No Symptoms of TMD”	*p*-Value
**AGE**			0.9502 ^1^
av. (SD)	24.98 (8.3)	24.88 (7.3)	
range	16.0–47.0	16.0–44.0	
median	21.75	22.7	
95%CI	[22.68;27.28]	[22.98;26.78]	
**SEX**			0.8630 ^2^
Female (%)	38 (76.0%)	41 (74.55%)	
Male (%)	12 (24.0%)	14 (25.45%)	

^1^ T-Student test; ^2^ Chi-square test.

**Table 5:** Distribution of skeletal classes between the examined groups.

Measurement	Study Group (n = 50) “Reciprocal Clicking”	Control Group (n = 55) “No Symptoms of TMD”	*p*-Value
Wits analysis [mm]			0.4680 ^1^
Skeletal class I (Wits = 0 ± 2 mm)	16 (32%)	23 (41.82%)	
Skeletal class II (Wits > 2 mm)	23 (46%)	24 (43.64%)	
Skeletal class III (Wits < −2 mm)	11 (22%)	8 (14.54%)	

^1^ Chi-square test.

**Table 6:** Assessment of the position and morphology of the mandible in both groups.

Measurement	Study Group (n = 50) “Reciprocal Clicking”	Control Group (n = 55) “No Symptoms of TMD”	*p*-Value
**SNPg angle [°]**			0.1933 ^1^
av. (SD)	78.23 (3.67)	79.14 (3.32)	
range	70.0–86.0	73.0–88.0	
median	78.25	79.0	
95%CI	[77.21;79.25]	[78.26;80.01]	
**NSa angle [°]**			0.8956 ^1^
av. (SD)	124.0 (4.9)	123.9 (5.0)	
range	114.5–135.0	113.0–134.0	
median	123.8	123.0	
95%CI	[122.6;125.4]	[122.5;125.2]	
**SaGo angle [°]**			0.2926 ^1^
av. (SD)	145.6 (7.4)	144.0 (7.7)	
range	122.5–165.0	128.5–161.0	
median	145.0	144.0	
95%CI	[143.5;147.7]	[141.9;146.1]	
**FMA [°]**			0.7698 ^1^
av. (SD)	25.3 (4.9)	24.9 (5.7)	
range	12.50–35.0	8.0–41.5	
median	25.0	25.0	
95%CI	[23.9;26.6]	[23.4;26.5]	
**NL/ML [°]**			0.4340 ^1^
av. (SD)	23.2 (6.3)	22.3 (5.9)	
range	10.5–38.5	7.0–35.0	
median	23.5	22.5	
95%CI	[21.4;25.0]	[20.7;23.8]	
**aGoGn angle [°]**			0.6575 ^1^
av. (SD)	122.0 (6.7)	122.6 (7.1)	
range	108.0–134.5	100.5–136.5	
median	123.5	122.5	
95%CI	[120.1;123.9]	[120.7;124.5]	
**aGoN angle [°]**			0.0629 ^1^
av. (SD)	49.5 (3.9)	51.0 (4.4)	
range	41.0–57.5	42.5–59.0	
median	49.8	51.5	
95%CI	[48.4;50.6]	[49.9;52.2]	
**NGoGn angle [°]**			0.3604 ^1^
av. (SD)	72.5 (5.3)	71.6 (5.1)	
range	61.5–83.0	55.5–84.0	
median	72.0	71.5	
95%CI	[71.0;74.0]	[70.2;72.9]	

^1^ T-Student test.

**Table 7:** Assessment of the position of the mandibular incisors in both groups.

Measurement	Study Group (n = 50) “Reciprocal Clicking”	Control Group (n = 55) “No Symptoms of TMD”	*p*-Value
**FMIA angle [°]**			0.6581 ^1^
av. (SD)	61.4 (7.9)	60.7 (8.5)	
range	45.5–76.5	42.5–85.0	
median	60.5	60.0	
95%CI	[59.2;63.7]	[58.4;63.0]	
**IMPA angle [°]**			0.5494 ^1^
av. (SD)	93.3 (7.6)	94.3 (9.5)	
range	76.0–106.5	75.0–121.0	
median	94.0	95.0	
95%CI	[91.2;95.5]	[91.8;96.9]	
**L1/Occl_Md_ angle [°]**			0.6349 ^2^
av. (SD)	71.2 (7.8)	70.1 (8.8)	
range	59.0–85.5	47.0–92.5	
median	70.5	68.5	
95%CI	[69.0;73.4]	[67.8;72.5]	
**L1/U1 angle [°]**			0.5347 ^1^
av. (SD)	134.9 (15.6)	133.0 (14.7)	
range	95.0–168.5	88.5–169.5	
median	133.3	129.5	
95%CI	[130.4;139.3]	[129.1;137.0]	

^1^ T-Student test; ^2^ U Mann‒Whitney test.

## Data Availability

The data underlying this article are available in the article.
